# Intracoronary infusion of mononuclear cells after PCI-treated myocardial infarction and arrhythmogenesis: is it safe?

**DOI:** 10.1007/s12471-012-0251-4

**Published:** 2012-02-14

**Authors:** L. F. H. J. Robbers, R. Nijveldt, A. M. Beek, M. J. B. Kemme, R. Delewi, A. Hirsch, A. M. van der Laan, P. A. van der Vleuten, J. J. Piek, F. Zijlstra, A. C. van Rossum, On behalf of the HEBE investigators

**Affiliations:** 1 Department of Cardiology, VU University Medical Center, ZH 5 F003, PO Box 7057, 1007 MB Amsterdam, the Netherlands; 2 Interuniversity Cardiology Institute of the Netherlands (ICIN), Utrecht, the Netherlands; 3 Department of Cardiology, Academic Medical Center, Amsterdam, the Netherlands; 4 Department of Cardiology, University Medical Center Groningen, University of Groningen, Groningen, the Netherlands; 5 Department of Cardiology, Erasmus University Medical Center, Rotterdam, the Netherlands

**Keywords:** Acute myocardial infarction, Cardiac arrhythmia, Cardiovascular magnetic resonance imaging, Severe arrhythmogenic events, Cell therapy

## Abstract

To reduce long-term morbidity after revascularised acute myocardial infarction, different therapeutic strategies have been investigated. Cell therapy with mononuclear cells from bone marrow (BMMC) or peripheral blood (PBMC) has been proposed to attenuate the adverse processes of remodelling and subsequent heart failure. Previous trials have suggested that cell therapy may facilitate arrhythmogenesis. In the present substudy of the HEBE cell therapy trial, we investigated whether intracoronary cell therapy alters the prevalence of ventricular arrhythmias after 1 month or the rate of severe arrhythmogenic events (SAE) in the first year. In 164 patients of the trial we measured function and infarct size with cardiovascular magnetic resonance (CMR) imaging. Holter registration was performed after 1 month from which the number of triplets (3 successive PVCs) and ventricular tachycardias (VT, ≥4 successive PVCs) was assessed. Thirty-three patients (20%) showed triplets and/or VTs, with similar distribution amongst the groups (triplets: control *n* = 8 vs. BMMC *n* = 9, *p* = 1.00; vs. PBMC *n* = 10, *p* = 0.67. VT: control *n* = 9 vs. BMMC *n* = 9, *p* = 0.80; vs. PBMC *n* = 11, *p* = 0.69). SAE occurred in 2 patients in the PBMC group and 1 patient in the control group. In conclusion, intracoronary cell therapy is not associated with an increase in ventricular arrhythmias or SAE.

## Introduction

Although the short-term mortality after acute myocardial infarction has decreased over the last decades, long-term morbidity remains high due to congestive heart failure caused by post-infarction remodelling [1–3]. To reduce the burden of chronic illness, new adjuvant treatment options are being explored to attenuate these adverse processes. Several experimental studies have suggested that cell therapy may improve functional recovery in patients after ST-elevation myocardial infarction (STEMI) due to induction of neoangiogenesis and beneficial paracrine effects, limiting adverse processes such as inflammation and remodelling [4]. More recent studies have suggested that certain subsets of (bone marrow derived) mononuclear cells contribute to the repair by stimulating the production of new cardiomyocytes from endogenous progenitor cells [5]. However, critics of cell therapy have expressed their concern regarding the safety of cell therapy, claiming that this technique may be applied too quickly, without extensive knowledge of all the possible effects of cell therapy. Specifically, fear has been expressed regarding the potential arrhythmogenic effects as was seen with the use of skeletal myoblasts for cell therapy in patients with ischaemic cardiomyopathy [6]. Multiple pro-arrhythmogenic properties of cell therapy have been proposed, such as the induction of tissue inhomogeneities, abnormal intercellular electrical coupling and autonomic function, leading to pathways for slow and/or unidirectional conduction [7, 8]. These unwanted effects of cell therapy may form a substrate for the development of ventricular arrhythmias, since it can provide the necessary conditions for arrhythmogenesis (i.e. a tissue substrate to form a reentry pathway, triggering factors and facilitating conditions) [9].

Between August 2005 and April 2008, the HEBE trial was conducted to assess the effect of intracoronary infusion with autologous mononuclear cells derived from either bone marrow (BMMCs) or peripheral blood (PBMCs), compared with standard therapy on recovery of regional and global left ventricular function after a revascularised acute myocardial infarction. The trial did not show an effect of either BMMCs or PBMCs on functional improvement at short-term follow-up [10]. To evaluate the safety of cell treatment, this substudy investigates the effect of intracoronary infusion of BMMCs or PBMCs after revascularised acute myocardial infarction on the prevalence of ventricular arrhythmias after 1 month and the occurrence of severe arrhythmogenic events (SAE) in the first year.

The study design of the HEBE trial has been reported in detail previously [11]. In short, patients between 30 and 75 years of age with a first STEMI treated with primary PCI were included in this multicentre trial. Between 3 and 7 days after PCI, patients underwent cardiovascular magnetic resonance imaging (CMR). After successful CMR imaging, patients were screened for eligibility and signed informed consent. Participating patients were randomly assigned in a 1:1:1 ratio to either additional intracoronary infusion of BMMCs, intracoronary infusion of PBMCs, or no cell therapy (i.e. only standard medical therapy). The study was conducted in accordance with the Declaration of Helsinki, and the study protocol was approved by the Institutional Review Boards of the participating institutes. Coronary angiography with subsequent intracoronary infusion of mononuclear cells was performed in patients randomised to BMMC and PBMC therapy, whereas no angiography or cell therapy was performed in patients randomised to the control group. The detailed cell processing and cell characterisation protocols have been reported previously [11]. A 24-hour Holter registration was performed 1 month after PCI, together with a resting ECG. Clinical follow-up was obtained at 1 month, 4 months and 1 year after the index event. For this substudy, SAE was defined as any event consisting of either sudden cardiac death or documented ventricular arrhythmias for which external defibrillation or ICD therapy was required.

## Data acquisition and analysis

In all participating patients, CMR was performed at least 48 h after PCI in a clinical 1.5 Tesla MR scanner with the use of a phased-array cardiac receiver coil. Functional imaging was performed by using ECG-gated steady-state free precession cine imaging with breath-holding, for the acquisition of short-axis images covering the entire left ventricle (i.e. from base to apex). From these images, left ventricular volumes were measured and ejection fraction (LV EF) calculated [11]. Late gadolinium enhancement (LGE) images were acquired 10–15 min after administration of a gadolinium-based contrast agent (Dotarem, Guerbet, Villepinte, France), using a 2-dimensional segmented inversion recovery gradient-echo pulse sequence, with similar full short-axis coverage of the left ventricle. Using the standard deviation (SD) method with a threshold window setting of 5 SD above the average signal intensity of unaffected myocardium, total infarct size was quantified [12]. Analyses were performed with dedicated software (MASS v.5.1 2010-EXP beta, Medis, Leiden, the Netherlands) and the performing analysts were blinded to the patient data during the analyses.

One month after PCI, 24-hour Holter registration was performed. Analysis was done automatically by external core laboratories, including analyses of the rhythm and morphology of each individual complex (supraventricular, AV nodal or ventricular). Results were manually verified. The arrhythmias were defined as follows: triplet PVCs were defined as a series of 3 successive ventricular complexes with a maximal RR interval of less than 600 ms (i.e. frequency of >100/min). Ventricular tachycardia (VT) was defined as any series of 4 or more successive ventricular complexes with a maximal RR interval of less than 600 ms (i.e. frequency of >100/min). By correcting for the total recording time, the mean number of triplets and/or ventricular tachycardias per 24 h for each patient was calculated for standardisation. Together with the Holter registration, a resting 12-channel electrocardiogram (ECG) was made from which the QRS width and corrected QT time (QTc) were measured.

Statistical analyses were performed on the basis of the intention-to-treat principle. Tests were performed using the Standard Package for the Social Sciences (SPSS 15.0).

## Results

Of the initial 200 patients, paired sets of CMR data and Holter data were available in 164 patients, with equal distribution amongst the treatment groups (BMMC *n* = 60, PBMC *n* = 53, control group *n* = 51). No differences were found in the baseline characteristics between the treatment groups for infarct size, LV volumes or LV function (Table 1). Likewise, electrolyte levels and the duration of the QT interval were similar in the three groups (Table 1).

**Table 1:** Functional parameters, infarct size and arrhythmia parameters of the three treatment groups

Characteristic	Total group (*n* = 164)		BMMC (*n* = 60)		PBMC (*n* = 53)		Control (*n* = 51)		BMMC vs. Control	PBMC vs. Control
									p-value	p-value
Functional and infarct mass parameters										
Days between primary PCI and MRI	3(3–4)		3(2–4)		3(3–4)		3(3–5)		0.79	0.81
End-diastolic volume (ml•m-2)	98 ± 16		97 ± 14		98 ± 16		100 ± 17		0.39	0.50
End-systolilc volume (ml•m-2)	57 ± 15		55 ± 15		57 ± 15		59 ± 15		0.20	0.37
Left ventricular ejection fraction (%)	43 ± 9		44 ± 9		43 ± 8		41 ± 8		0.14	0.37
Infarct mass (% of LV mass)	19% ± 9%		19% ± 10%		18% ± 9%		20% ± 10%		0.57	0.30
	
Arrhythmia parameters										
Duration of Holter (hours) at 1 month	24(23–25)		24(23–25)		24(23–24)		24(23–26)		0.76	0.052
QRS duration (ms) at 1 month	94(86–100)		92(83–100)		94(87–100)		96(88–100)		0.08	0.32
Corrected QT interval (QTc) at 1 month	420 ± 27		418 ± 29		418 ± 28		423 ± 24		0.37	0.33
Serum sodium level (mMol•l-1) at 1 month	141 ± 2		141 ± 2		141 ± 2		142 ± 2		0.40	0.01
Serum potassium level (mMol•l-1) at 1 month	4.3 ± 0.3		4.4 ± 0.4		4.3 ± 0.3		4.3 ± 0.3		0.55	0.87
Class II anti-arrhythmic drug use during Holter (%)	157	96%	55	92%	49	92%	50	98%	0.22	0.36
	
Patients with triplet PVCs (%)	27	16%	9	15%	10	19%	8	16%	0.92	0.67
Patients with ventricular tachycardias (>4 PVCs) (%)	29	18%	9	17%	11	21%	9	15%	0.70	0.69
Patients with both triplet PVCs and ventricular tachycardias (%)	13	8%	4	7%	6	11%	3	6%	1.00	0.49
No. of triplet PVCs/24 h (when present)	1	(1–3)	2	(1–4)	1	(1–1)	2	(1–3)	0.74	0.42
No. of ventricular tachycardias (when present)	1	(1–3)	1	(1–3)	2	(1–3)	1	(1–3)	0.69	0.73

BMMC = Bone marrow-derived mononuclear cells, PBMC = peripheral blood-derived mononuclear cells, PCI = percutaneous coronary intervention. P-values are calculated by comparing treatment group vs. control. Data presented as mean ± standard deviation or as median (interquartile range)

**Fig. 1:**
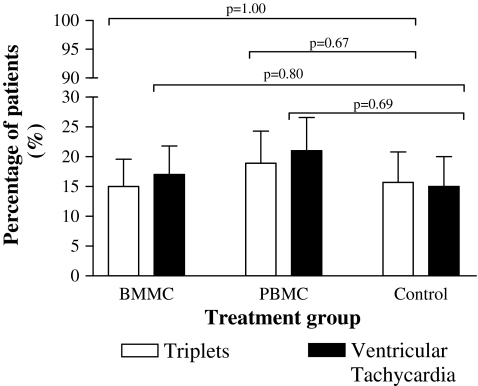
The prevalence of triplets and ventricular tachycardias between the treatment groups. BMMC = Bone Marrow-derived Mononuclear Cells, PBMC = Peripheral Blood-derived Mononuclear Cells, PCI = Percutaneous coronary intervention. P-values are calculated by comparing treatment group vs. control

During the Holter registration at 1 month, 33 patients showed either triplet PVCs, ventricular tachycardia or both. Distribution was similar amongst the treatment groups for both triplets (control *n* = 8 vs. BMMC *n* = 9, *p* = 1.00; vs. PBMC *n* = 10, *p* = 0.67) and ventricular tachycardias (control *n* = 9 vs. BMMC *n* = 9, *p* = 0.80; vs. PBMC *n* = 11, *p* = 0.69). In these 33 patients, a median of 1 triplet (interquartile range, IQR 1–3) per 24 h and a median of 1 ventricular tachycardia (IQR 1–3) occurred during registration. Again, no differences existed between the treatment groups (Fig. 1).

Of all 200 patients, 9 suffered from a severe arrhythmogenic event during the first year of follow-up. One patient assigned to the PBMC group died of VF during the first year of follow-up, 13 days after cell therapy; autopsy revealed an in-stent thrombosis. Two patients had documented VF with successful resuscitation without sequelae and received an ICD implantation. One of these patients was also assigned to the PBMC and suffered from VF a few hours after cell infusion. The other patient was assigned to the control group and VF occurred 3 days after randomisation. Additionally, 6 patients received an ICD for primary prevention of late arrhythmias due to a low EF as indicated by the guidelines of the European Society of Cardiology [13]. No cases of appropriate ICD discharge in these patients have been reported in the first year.

## Discussion

This HEBE trial substudy focused on the safety of cell therapy with mononuclear cells after a revascularised STEMI, with regard to arrhythmogenesis and the prevalence of SAE, defined as either sudden cardiac death or documented ventricular arrhythmias for which external defibrillation or ICD therapy was required. One month after PCI and cell therapy, the amount of triplet PVCs or ventricular tachycardias are similar between the three treatment groups. Secondly, no significant differences were found in the rate of severe arrhythmogenic events in the first year of follow-up. This shows that the current method of cell therapy, i.e. intracoronary infusion of mononuclear cells, does not harbour an increased risk of arrhythmogenesis. The choice to discriminate between triplet PVCs and ventricular tachycardia was made in line with the results of the recently published MERLIN-TIMI 36 substudy. This study demonstrated that ventricular arrhythmias of 4 beats or more are associated with worse prognosis [14]. Our findings are in concordance with earlier cell therapy trials that did not report an increase in arrhythmias or adverse events either while using similar cell types and techniques as the HEBE trial [15, 16]. Vice-versa, the contradictions with earlier findings regarding increased arrhythmogenesis after cell therapy are most likely due to the use of a different cell type [6, 17, 18]. For instance, these studies used skeletal myoblasts instead of mononuclear cells from either bone marrow or peripheral blood. An animal study by Leobon et al. showed that transplanted skeletal myoblasts do not show electrical coupling with cardiomyocytes after differentiation and are functionally isolated from the surrounding tissue [8]. Myoblasts differentiate locally in cardiomyocyte-like cells, which are electrically active, whereas mononuclear cells are not electrically active and promote repair by promoting neoangiogenesis and endogenous repair by local progenitor cells [4, 5]. Furthermore, the skeletal myoblasts were grafted directly into the myocardial scar by intramyocardial injection during CABG, whereas the HEBE trial used intracoronary infusion of the revascularised infarct-related artery. Theoretically, the intramyocardial injection is more reliable in ensuring that the stem cells reach the intended target, but it could also lead to a more pronounced disruption of the tissue structure due to secondary myocardial damage associated with direct injection of cell cultures.

An important issue that needs to be addressed is that no positive effects of cell therapy have been found for the main endpoints of the HEBE trial [10]. Therefore, it remains unclear whether cell therapy has any influence on human myocardial tissue in a clinical setting. The main results of the HEBE trial demonstrate that cell therapy has no additional effect on the recovery of myocardial function after a revascularised acute myocardial infarction [10]. Recently, a HEBE trial flow-Doppler substudy showed that the method of cell therapy did not improve the microcirculation either [19]. It is possible that cell therapy with mononuclear cells has more subtle effects on the recovery processes after an acute myocardial infarction than recovery of systolic function. As is known from theory of the ischaemic cascade, many adverse processes (e.g. metabolic changes, impaired perfusion and impaired diastolic function) precede the disruption of systolic function [20]. It was recently shown that a subpopulation of bone marrow derived mononuclear cells (C-Kit+ cells) may have an additional effect on the endogenous repair processes by stimulating local progenitor cells [5]. The amount of C-Kit+ cells within the administered cell suspensions that were used in the HEBE trial is not known. Secondly, the role of C-Kit+ cells in myocardial repair after AMI in humans is still unknown. Future research on different subpopulations of mononuclear cells and their ability to induce neovascularisation, attenuating the inflammatory response or stimulating local progenitor cells, all essential parameters for determining whether functional improvement is needed in order to appreciate a broader range of effects of mononuclear cell therapy in humans.

## Conclusion

Cell therapy by intracoronary infusion of mononuclear cells is not associated with either an increase or decrease in the amount of triplets or ventricular tachycardias. Secondly, no increased incidence in severe arrhythmogenic events was found in the first year of follow-up. Therefore, cell therapy consisting of intracoronary infusion of mononuclear cells does not impose additional dangers with regard to ventricular arrhythmias.
